# The American Dental Weekly

**Published:** 1897-09

**Authors:** 


### The American Dental Weekly.

The *American Dental Weekly*, B. H. Catching, editor and publisher, the first number to be issued September 2d, 1897, at Atlanta, Ga.

This is the only dental weekly in this country if not in the world. This journal will certainly not be crowded. It may possibly be a little lonely while it is in its swaddling clothes, but Brother Catching is a good nurse and will take excellent care of the infant till it attains strength. We bespeak for the *Weekly* all needed aid and success, and may it early grow to a robust manhood.

